# Simple Fall Criteria for MEMS Sensors: Data Analysis and Sensor Concept

**DOI:** 10.3390/s140712149

**Published:** 2014-07-08

**Authors:** Alwathiqbellah Ibrahim, Mohammad I. Younis

**Affiliations:** 1 Department of Mechanical Engineering, State University of New York at Binghamton, Binghamton, NY 13902, USA; E-Mail: aibrahi4@binghamton.edu; 2 Department of Mechanical Engineering, King Abdullah University of Science and Technology (KAUST), Thuwal 23955-6900, Saudi Arabia

**Keywords:** fall detection, MEMS switches, sensing algorithm

## Abstract

This paper presents a new and simple fall detection concept based on detailed experimental data of human falling and the activities of daily living (ADLs). Establishing appropriate fall algorithms compatible with MEMS sensors requires detailed data on falls and ADLs that indicate clearly the variations of the kinematics at the possible sensor node location on the human body, such as hip, head, and chest. Currently, there is a lack of data on the exact direction and magnitude of each acceleration component associated with these node locations. This is crucial for MEMS structures, which have inertia elements very close to the substrate and are capacitively biased, and hence, are very sensitive to the direction of motion whether it is toward or away from the substrate. This work presents detailed data of the acceleration components on various locations on the human body during various kinds of falls and ADLs. A two-degree-of-freedom model is used to help interpret the experimental data. An algorithm for fall detection based on MEMS switches is then established. A new sensing concept based on the algorithm is proposed. The concept is based on employing several inertia sensors, which are triggered simultaneously, as electrical switches connected in series, upon receiving a true fall signal. In the case of everyday life activities, some or no switches will be triggered resulting in an open circuit configuration, thereby preventing false positive. Lumped-parameter model is presented for the device and preliminary simulation results are presented illustrating the new device concept.

## 1. Introduction

Several algorithms and sensor concepts have been introduced over the past few years for fall detection of the elderly. These have been based on the available theoretical and experimental kinematical data for falling of humans during various kinds of falls as well as during everyday life activities ADL. With the advances of micromachining technologies, and in order to successfully develop sensors based on microelectromechanical systems (MEMS) technology, there is a need for more detailed studies and precise data geared for the requirements of MEMS. Directionality of motion and acceleration components is crucial for MEMS structures, especially those relying on electrostatic transduction.

Falls can be defined as unintentionally coming to the ground or a lower level as a consequence of sustaining a violent blow, loss of consciousness, sudden onset of paralysis such as in a stroke or an epileptic seizure [1]. Falling onto the ground is one of the most common problems that may cause serious injuries to the human body, especially for the elderly; it can sometimes be fatal [2]. In addition, the medical treatment of the elderly faller who has fallen and remained on the ground for a long time is very costly. Getting immediate help after the fall can decrease the risk of palsy and death [3–6]. The impact of falls on the elderly is considered to be the sixth cause of death for people aged 65 and the main cause of death for those over 75 years old [7]. Thirty-two percent of the elderly over 75 years of age have fallen at least once a year, and among them, 24% have been seriously injured [8,9]. The rate of falls increases significantly among elderly people living in nursing homes, with at least 40% of the patients falling two or more times within 6 months [10].

People who suffer from Alzheimer's, Parkinson's and chronic diseases are more likely to fall [10]. Falls associated with a loss of consciousness, such as syncope, stroke, and epileptic seizure are not easily recognized [11]. Seniors who are living by themselves may lose consciousness during a fall and may have a disease, such as Alzheimer's, that prevent them from asking for help or describing where they are [12]. Non-automatic alarm systems in such scenarios do not work. Even in the case of an elderly person who is still conscious, they may panic after the fall and forget to press the alarm button.

The consequences of falls can lead to fractures and in some cases, death. Hip, wrist, fractures of the elbow and forearm and head injuries are common due to falls. A fall also involves hidden damages that affect the self-confidence of a person [3,4]. Common consequences are fear, loss of independence, limited capabilities, low self-esteem and generally, a lower quality of life. Even if there are no immediate consequences, the long-wait on the floor for help may lead to additional health problems such as hypothermia, confusion, complications, and even death [3]. If a person falls and cannot get assistance within an hour, severe complications can arise resulting in fatalities within six months [5]. If an elderly or disabled person can get help immediately after the fall, the severity of the injury can be mitigated. It also results in decreasing the risk of paralysis and death [6].

Therefore, immediate and reliable real-time methods of detections are required so that adequate medical support can be provided in time. There has been increasing interest in the past couple of decades on developing systems for the detection of falls for the elderly [2–27]. These systems are based on employing sensors, accelerometers and/or gyroscopes, to detect changes in the dynamics features or posture of the body during falling. Algorithms are then developed and utilized to distinguish the signals of falling from the ADL. Most algorithms are based on a threshold value from the maximum peak resultant acceleration or other related parameters, such as the sum-vector of acceleration in the falling plane, sum-vector of velocity of all spatial components right before the impact, and the sum-vector of acceleration of all spatial components. Other parameters such as the change in orientation or posture detection after the fall have been also investigated [6]. One primary aim of the present study is to propose a switch-based fall detection system that avoids the use of complex algorithms and processors. This simplification will result in a device that is more practical, reliable, robust, and better suited to wide-scale use.

Different types of sensors were used, like accelerometers and gyroscopes, and the focus was on the total acceleration or total velocities without taking into account the effect of each component alone. Among the first to conduct research in this field were Williams *et al.* [7] who proposed the design of a smart sensor to detect falls and monitor activity and discussed its integration within an intelligent telecare system. The proposed device measures the impacts associated with a fall and monitors the status of the faller to identify whether assistance is required. Since then, several studies followed focusing on monitoring physical activities to be able to detect body postures (sitting, standing, lying, and falling) through use of accelerometers and a combination of accelerometers and gyroscopes [13–18]. Sandler *et al.* [28] modeled the human body into one-, two-, and three-link models and focused on the kinetic energy and impact velocity of the body during the fall. Nam *et al.* [29] modeled the human body as a 2DOF mass-spring-damper system to measure the reaction force on the hand when the faller hits the ground. Hwang *et al.* [8] developed an algorithm and real-time monitoring ambulatory system using a Bluetooth module for fall detection in the elderly using an accelerometer, gyroscope and tilt sensor. Lindemann *et al.* [11] discussed a fall detector based on accelerometers and placed at head level as well as an algorithm to distinguish between ADL and simulated falls. Tamura [23] investigated wearable monitor that the patient carries at the hip, which is comprised of a tri-axial accelerometer, microcomputer and data logger. Chen *et al.* [12] designed a fall detection sensor worn at the waist using a tri-axial accelerometer in conjunction with a wireless network. They also observed the acceleration from the backward fall and compared it to sitting and walking activities. They reported some overlap between these activities and falls. Bourke *et al.* [19] distinguished between falls and ADLs using a bi-axial gyroscope worn at the chest and specified the required thresholds of inclination, angular velocity and angular acceleration to recognize a fall. Shi *et al.* [30] developed an airbag protection technique to save the hip from fractures using a MEMS sensor that detects the fall and triggers the inflation of the airbag just before the impact. Tan *et al.* [31] compared the impact velocities of the wrist between the backward fall and forward fall. Bourke *et al.* [22] simulated fall events and ADL for the trunk and thigh using a tri-axial accelerometer.

Marschollek *et al.* [24] provided three ways to assess and classify the data collected from the accelerometers worn at the waist of the faller into high or low risk group fallers. Wang *et al.* [21] tried to increase the sensitivity of the fall detection by placing tri-axial accelerometers on the head above the ear. Zhang *et al.* [10] focused on the detection of falls and motionless activities such as standing, sitting and lying using waist worn box of accelerometer, gyroscope and magnetometer sensors to distinguish between falls and ADLs.

Li *et al.* [20] used accelerometers and gyroscopes on the chest and thigh to analyze falls and daily living activity. Total linear acceleration and rotational rate thresholds were used to recognize a fall. Jantaraprim *et al.* [6] used a tri-axial accelerometer fixed at the trunk to distinguish between falls and daily activities based on the total acceleration. They indicated that more sophisticated algorithms for the discrimination of falls from normal activities in the elderly are needed to improve performance of detection. Perry *et al.* [4] evaluated the existing fall detection approach to show the most effective method between using accelerometers alone or multi sensors together. They found that the acceleration of the faller played a critical role in the faller's motion and that it should be used for fall detection. Tomkun *et al.* [32] designed a fall detection system based on accelerations threshold extracted from experimental falls and daily activities using a bi-accelerometer and gyroscope placed at the chest. Bourke *et al.* [25] extracted the velocity threshold and acceleration from the RSS with upper and lower thresholds using a waist worn tri-axial accelerometer based on the minimum peak values from all falls. Three different parameters associated with falls were examined: velocity, impact and posture. Abbate *et al.* [2] designed a sensor nodes network of accelerometers and gyroscopes to collect the data and send it to a base station, which analyzes the data and sends a fall alarm to the paramedics in the case of serious falls. Lai *et al.* [26] used multiple tri-axial accelerometers worn at different positions on the body such as left hand, right hand, body, left foot, and right foot to collect the acceleration and inclination data that used in specifying thresholds.

Finally, the use of smartphones with embedded accelerometers has been recently proposed for fall detection [27]. A major problem associated with this is the mobility of the cell phone (it is not mounted in a fixed position), which complicates the calibration and results in significant numbers of false alarms. Also, a trivial fall detection algorithm is used causing many false positives. To avoid false positives, the user should press a button to dismiss the alarm when there is no real fall [2].

As noted from the above review, there are numerous systems that rely on the use of signal processing algorithms that process data from one or several sensors. Relying on multiple sensors (gyroscopes, accelerometers), multiple node locations for posture determination (knees, waist, trunk, head), and use complex algorithms for fall thresholds improves the sensitivity, however it raises costs and adversely affects the practicality of the systems. Most systems need an additional decision unit to trigger an alarm. It is desirable to have a reliable detector that is simple, small in size, convenient to use and wearable, and consumes low power, which fit the features that the MEMS technology offers. Hence, we aim to explore the possibility of using smart switches based on the MEMS technology, for fall detection. These switches are designed to be triggered only due to a true fall signal. Toward this goal, precise data on the variation of the acceleration components on the human body in all directions during falls and ADLs need to be analyzed. Currently, however, there is a lack of such data. Most of the available data display the Root-Sum-Squares (RSS) of all three acceleration components and not each individual one. Such information is critical for MEMS to enable proper design of a proof mass that moves in the desirable direction and to induce the required capacitive change upon fall.

Therefore, the objective of this paper is twofold: First, to provide detailed experimental data of acceleration components on the hip, chest and head, which are commonly proposed for sensor locations, for different kinds of falls and ADLs. We will analyze the obtained data, compare them to a simple 2-link model, and derive threshold criteria that could be used to recognize a fall based on uni-axial accelerometers. Second is to present a model and design guideline for building smart MEMS switches and their various configurations needed for fall detection.

In previous studies [33–36], the concept of a switch triggered by acceleration was investigated and demonstrated both theoretically and experimentally. The idea is to utilize the structural instability created by the combined effect of acceleration and electrostatic forces to force a microstructure to collapse, and thus, closing an electric circuit and activating a desirable event, such as to deploy an airbag in cars. In this study, we aim to explore utilizing similar concept for fall detection, which can simplify the fall detection algorithm through combining the functionality of sensing and actuation in one single device. In order to do that, however, detailed kinematic study needs to be conducted to reveal the exact changes in acceleration, in both magnitude and direction, which occur upon falling for various node locations on the human body.

The organization of the rest of the paper is as follows: in Section 2, we discuss the modeling of the human body as two links model. We study the acceleration for hip, chest and head positions. Then, Section 3 deals with experimental work and analyzes the acceleration data collected from real fall for the three major positions: hip, chest and head. We provide proper fall thresholds for those positions that when exceeded the fall event can be recognized. Section 4 presents the MEMS switch design that will be triggered to detect the fall event according to the selected thresholds from the experimental data. Finally, in Section 5, we present a summary and conclusion of the paper.

## 2. Two DOF Model

**Figure 1.**
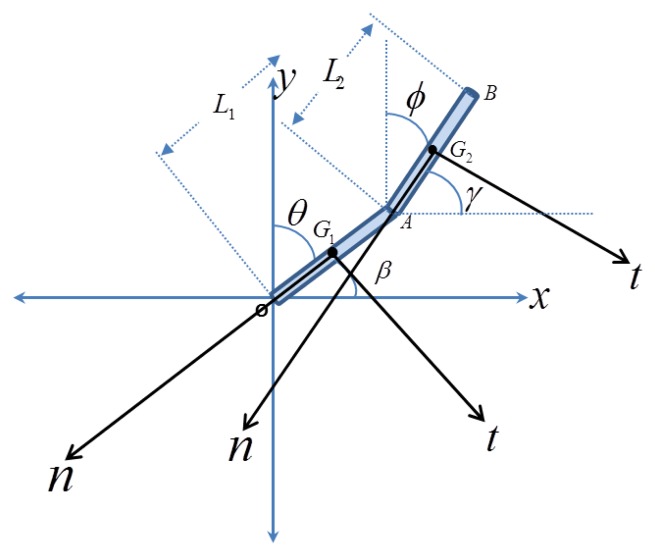
Two DOF model with points A, G2 and B representing the hip, chest and head positions, respectively.

Before proceeding to the experimental tests, it is helpful to consider a simple fall model that sheds light on the basic dynamical features of human during fall and also to give some guidance of how to interpret the experimental data. Toward this, a simple 2-link model (2-degree of freedom model) is adopted, Figure 1. Fall in this model is assumed to occur on a single plane of the page. The model assumes the human body as two rigid links, where points A, G2 and B represent the location of the hip, chest, and head, respectively. The local normal and tangential axes are drawn for each position to derive the local acceleration as a function of time. Using Lagrange equations, the equations of motion are derived (see Appendix A).

For a sensor installed on the human body, the acceleration components are written with respect to the local tangential and normal directions, *n* and *t*, with respect to either link *L*_1_ or link *L*_2_, as shown in Figure 1. These represent the acceleration that an actual accelerometer installed on these locations will detect. To simplify comparison with the experimental data, we add the static effect of gravity, which the proof mass of the accelerometers will feel. Note for example that for the initial position before the start of the fall when the proof mass of the accelerometer will be under the static effect of its own weight, hence it will indicate a value of +g in its reading even when there is no motion. Hence, we add this effect in the derived analytical expressions. The results are summarized in Table 1. where:

(1) $$a_{x}=(-L_{1}\ddot{\beta }\sin\beta -L_{1}{\dot{\beta }}^{2}\cos\beta )$$

(2) $$a_{y}=(L_{1}\ddot{\beta }\cos\beta -L_{1}{\dot{\beta }}^{2}\sin\beta )$$

*L*_1_ is the length of the $\bar{OA}$ link, *β* is the angle with *x*-axis, *β̇* and *β̈* are the angular velocity and angular acceleration, respectively.

**Figure 2.**
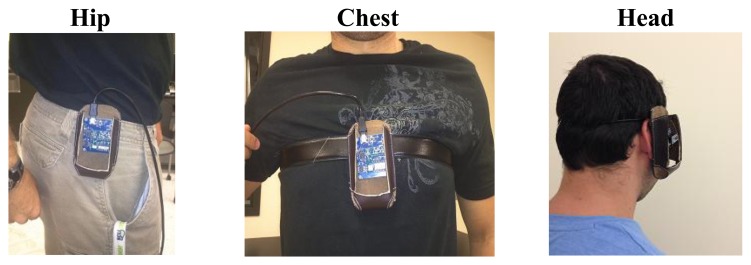
Sensor locations.

As a case study, the parameters of Table 2 are adopted for the two-link model of Appendix A, which along with the equations of Table 1 are used to obtain the acceleration components for each position. Normal and tangential acceleration for different kinds of falls for the three positions are summarized in Figure 2.

**Figure 3.**
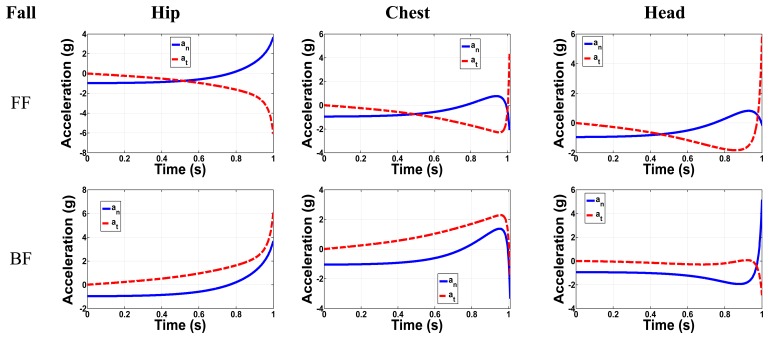
Forward Fall (FF) and Backward Fall (BF) accelerations for the hip, chest and head positions.

The acceleration is analyzed in all directions where the total time from starting to fall to the initial contact with the ground is around one second according to Chang *et al* [37]. Figure 3 shows the FF and BF normal and tangential accelerations for the three major positions: Hip, chest and head. It is clearly shown that in most cases the main difference between the FF and BF is in the direction of the tangential acceleration, while the maximum peaks have the same values. For FF in the hip position, the maximum acceleration in the normal and tangential direction is 4.7 g and 6 g, respectively. For the chest, the maximum FF normal and tangential accelerations were 1.8 g and 4.4 g, respectively. The tangential acceleration has a greater value than the normal one for the head in the FF case with a peak value of 6 g, while in the BF the normal acceleration is greater than the tangential one with a peak value of 5.9 g. We notice that the peak acceleration value increases as the position of the sensor changes from the lower level at hip to the higher one at head. In general the tangential acceleration for all positions exceeds 4 g, while the normal acceleration exceeds 2 g. These results are based on this simple 2-link model. They are not expected to be accurate; however they can be useful to understand the experimental data of the next section.

## 3. Experimental Data

A tri-axial accelerometer (MMA8450Q) by Freescale [38], with ±2 g/±4 g/±8 g dynamic selectable full scale is used to extract the accelerations of the human movements experimentally. The SENSOR TOOLBOX software is used to monitor the accelerations. The acceleration of the three axes for any fall or ADL is simulated. The sensor is fixed at different positions as shown in Figure 3. The effect of weight is taken into account by the designer of this sensor. This means that one of the axes has a value of −g or g based on the position of the sensor. The acceleration data are extracted for the real time ADL and falls in the three major positions: Hip, chest and head.

All activities are conducted by young volunteers for the safety of the elderly. A spring mattress is used to protect the faller from any injures. All measurements are repeated three times for each fall type or ADL. To represent the worst case scenario, the fall trials with minimum acceleration peak are used for analysis and simulation. Special Toolbox software is used to connect the sensor to a PC and to read the measured data. The volunteers who conducted the test were approximately of 85 kg weight, 1.8 m height and near 30 years of age.

**Figure 4.**
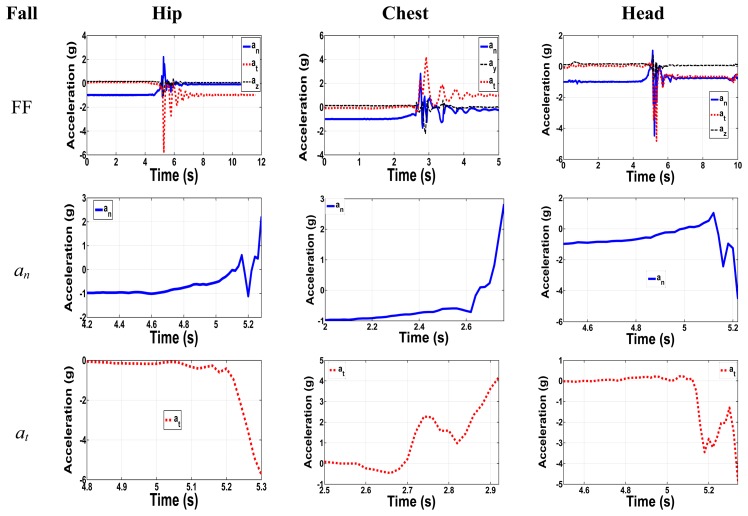
Samples of measured fall accelerations for the hip, chest and head positions.

Figure 4 shows the forward fall with zoom-in on the normal and tangential acceleration components for all positions. For the hip, the major components have maximum relative peak acceleration values of 3.2 g and 5.5 g. Although the normal component has a peak acceleration of 2.2 g, the total net change in g from the initial position with −1 g to the peak value of 2.2 g has absolute acceleration of 3.2 g. The out of plane component has the lowest acceleration value. Therefore, our focus will be on the other two major components, neglecting the out of plane one. The experimental results agree with the 2-link model results, since they have the same trend even though the peak values sometimes are different. As an example, if we compare the results for FF in Figure 2 and Figure 4 both normal and tangential components have the same trend. Normal component from Figure 2 start from −1 g to a higher positive peak of 3.7 g, while in Figure 4 the normal one start from −1 g to higher positive peak of 2.2 g. For tangential components in Figure 2 and Figure 4 they start from 0 g to lower negative peak values of −6 g and −5.7 g, respectively, which show same trend and peak values.

**Figure 5.**
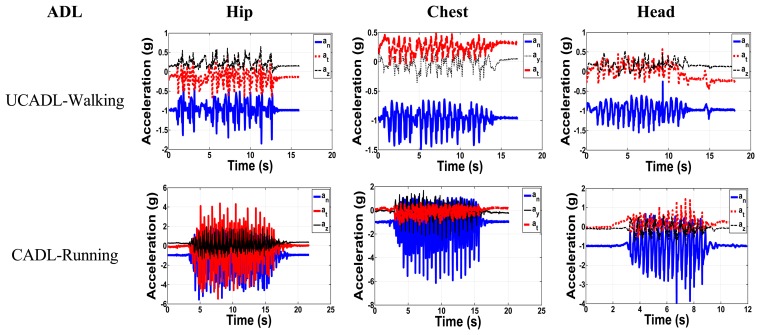
Samples of the CADL and the UCADL.

**Figure 6.**
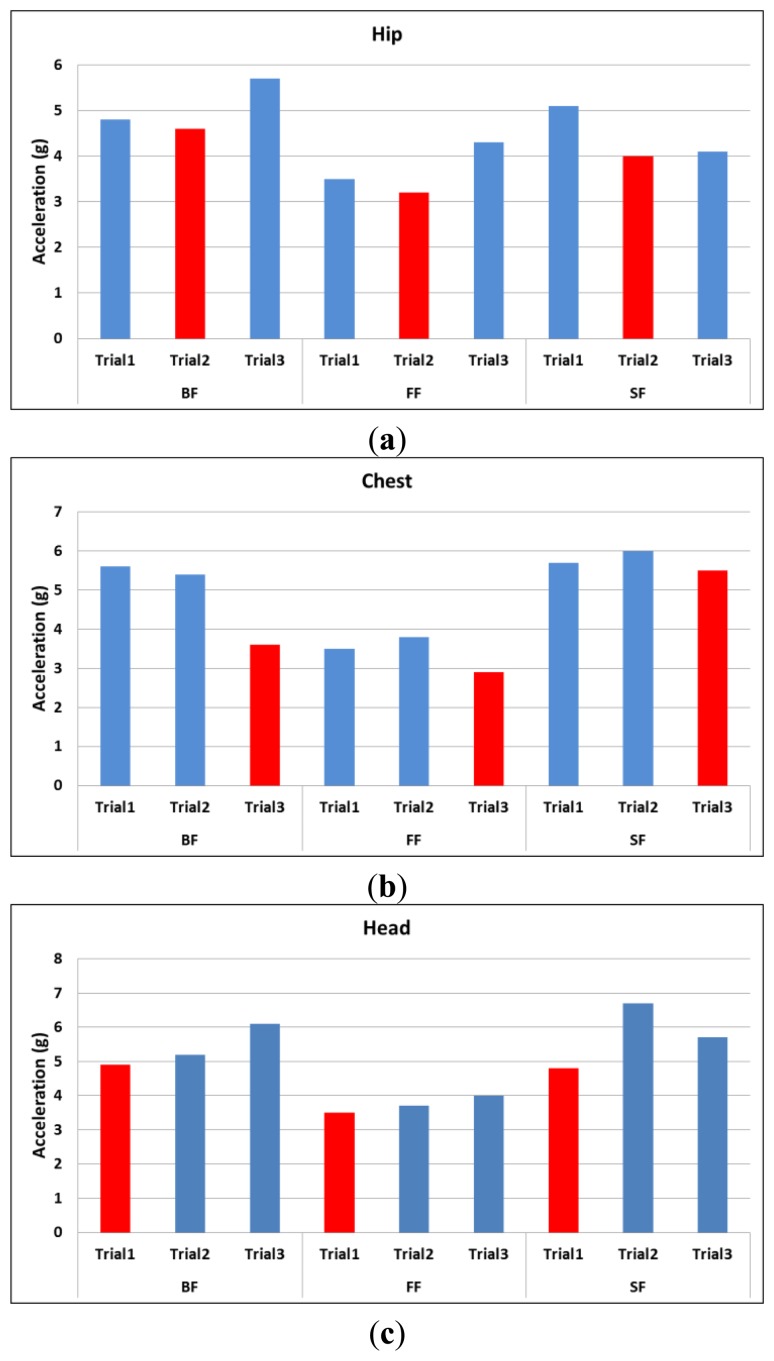
Peak values of three different trials in the normal direction for forward fall (FF), backward fall (BF) and side fall (SF) for the hip, chest and head positions.

It is very important to take into consideration the daily activity of the elderly and to monitor the acceleration components in each activity. We aim here to classify the daily activities into two major categories: The Critical Activities of Daily Living (CADL) and the Uncritical Activities of Daily Living (UCADL). CADL overlap with the acceleration peak values of falls, whereas UCADL do not. Samples of UCADL and CADL are shown in Figure 5 for the three positions. Figure 6 shows the UCADL (walking) with acceleration less than all fall levels in both normal and tangential components and the CADL (running), which overlap with the falls accelerations.

**Figure 7.**
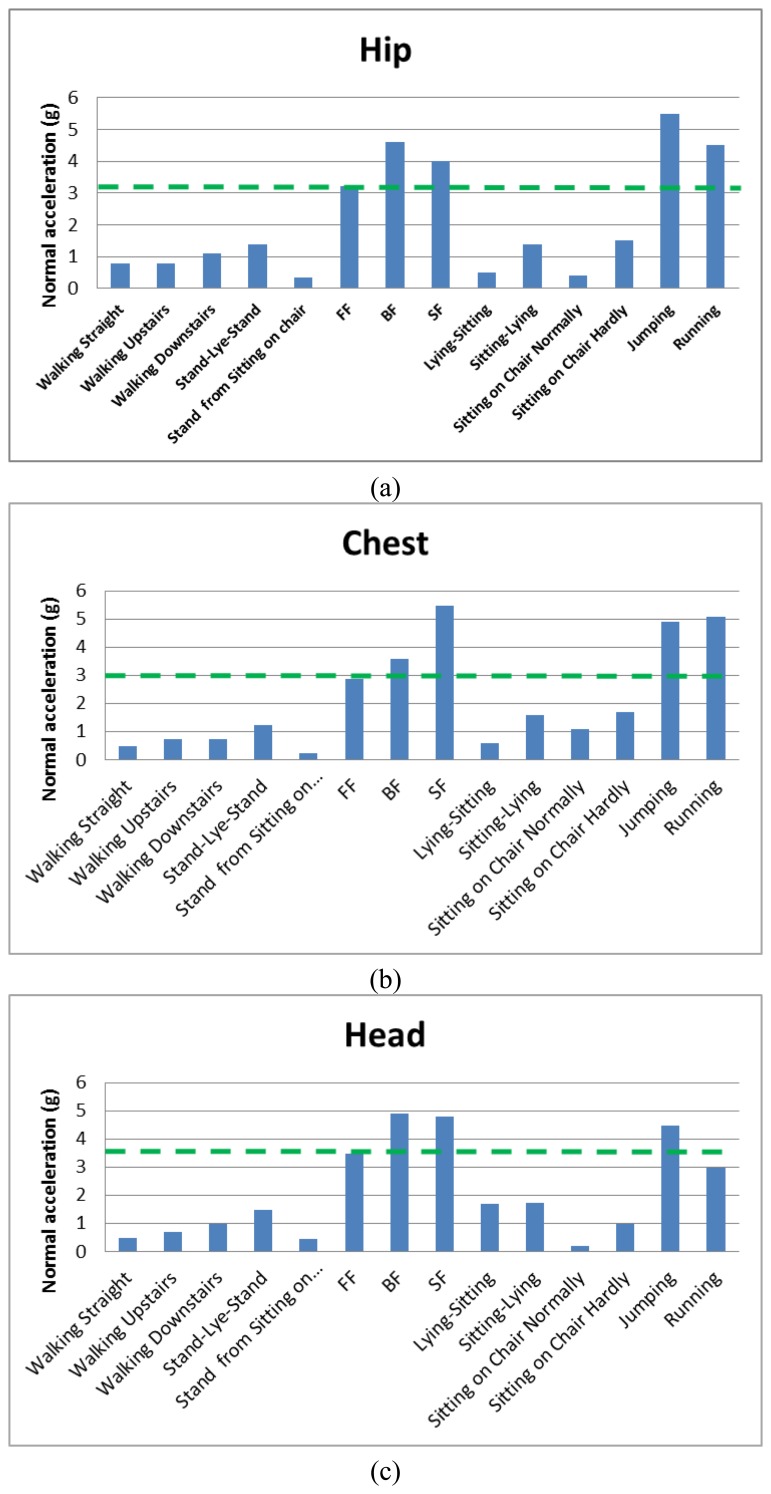
Measured normal acceleration of ADL and different kinds of falls: (**a**) hip; (**b**) chest; (**c**) head.

**Figure 8.**
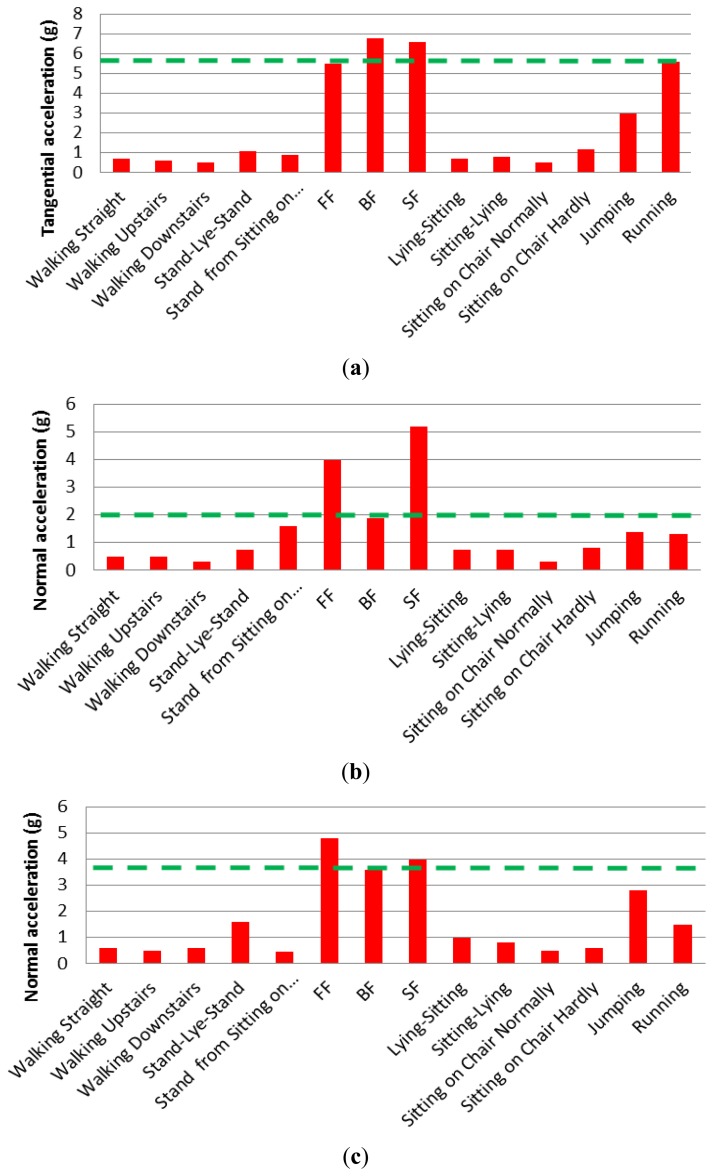
Measured tangential acceleration of ADL and different kind of falls: (**a**) hip; (**b**) chest; (**c**) head.

Figure 6 shows the peak values in the normal direction for three different trials of all types of fall. The minimum peak value among the three trials is labeled in red color. Since we are looking for acceleration peak values that may overlap with ADL, the trials labeled in red are taken to represent the worst case scenario as compared to the ADL in Figures 7 and 8.

Figure 7 shows the measured normal acceleration for different ADL and falls for different positions, while Figure 8 shows the measured tangential acceleration for different ADL and falls. In the hip position, the lowest normal acceleration among all falls is 3.2 g, while the lowest tangential fall acceleration is 5.5 g. In the chest position, the lowest in the normal direction is 1.9 g, while the tangential one has a value of 2.9 g. The head position has minimum fall values in the normal and tangential of 3.5 g and 3.6 g, respectively. Results shown in dashed green lines in Figure 7 and Figure 8, and summarized in Table 3, are the minimum values or the limits distinguishing between CADL and UCADL, as derived through the experimental data of Figures 7 and 8.

It is shown from Figures 7 and 8 that jumping and running are the most critical ADLs. In the hip position, the jumping activity exceeds the limit from Table 3 in the normal direction, but it is close to the limit in the tangential direction. While in the running activity, the acceleration peak exceeds both the normal and tangential limits. In the chest and head: jumping exceeds the limits in the tangential direction only, while in the running it exceeds the fall limit in the normal direction only. Under the assumption that elderly do not typically run or jump, these two activities are not taken into consideration here and they will be ignored when extracting the fall thresholds. Based on that, the fall thresholds are taken to be 85% of the acceleration signals of the fall experimental results. The reason for choosing this percentage is to be far enough from the ADL regime and close enough to the fall regime numbers. Table 4 shows all the extracted thresholds.

For any position (hip, chest, and head) without jumping and running, the maximum normal acceleration from ADL is 1.75 g, while the minimum normal fall acceleration is 2.9 g. Thus, the threshold for fall in the normal direction can be considered to be 2.3 g. The maximum tangential acceleration from ADL is 1.6 g, while minimum tangential fall acceleration is 1.9 g. Therefore, the threshold of fall can be assumed to be 1.9 g. According to fall signals, the backward fall of the hip position has higher acceleration peaks in normal and tangential directions of values 4.6 g and 6.8 g, respectively. Among daily activities, excluding jumping and running activities, stand-lie-stand activity of the head position has maximum normal and tangential acceleration peaks of 1.5 g and 1.6 g, respectively.

Next we introduce a unified limit that represents the maximum value of ADL. This will be derived based on correlating the minimum measured acceleration at fall to the maximum peak acceleration of the ADL. The minimum fall peaks and the maximum ADL peaks were in the normal direction. From the data in Figure 7 after excluding running and jumping activities, the approximated maximum ADL peaks in the normal direction were 1.5 g, 1.7 g, and 1.75 g for the hip, chest and head, respectively. On the other hand, the minimum normal acceleration peaks for the hip, chest and head were 3.2 g, 2.9 g and 3.5 g, respectively. As shown in Table 5 the maximum acceleration from ADL divided over the minimum acceleration from fall for each position, and the average of percentages were approximately equal 50%. This limit will be taken to represent the effect of ADL.

Table 6 shows the 50% value of the original fall amplitudes. For the hip position, the lowest limits in normal and tangential accelerations are 1.6 g and 2.75 g, respectively. For the chest position, the lowest values of the normal and tangential are 1.45 g and 0.95 g respectively. For the head position, the lowest limits are 1.75 g and 1.8 g in normal and tangential accelerations, respectively.

According to the above discussion, the maximum accelerations of the daily activities are considered much lower than the 50% of the maximum acceleration of the fall signals. In the next part, the 50% of the fall signal will be introduced to represent the ADL and to check the stability of the designed fall detection sensor under these signals, while the 85% of the fall signals will be introduced as the minimum threshold to trigger the switch indicating a fall. For example, the case of FF of the hip from Table 4 (*a_n_* = 2.7 g, *a_t_* = 4.7 g) should trigger the threshold sensor, while 50% of this signal (*a_n_* = 1.6, *a_t_* = 2.75 g) in Table 6, representing the ADL, will not trigger the threshold sensor.

Despite our attempt to extract reliable and accurate fall thresholds based on uni-axial accelerometers or single-axis acceleration measurements, the overlap between some of the ADL range with the thresholds of falls makes the likelihood of false positives considerable. To minimize this possibility, we propose the concept of using two threshold sensors, or switches. Each switch is sensitive to acceleration along either the tangential or normal direction of the falling plane. The fall criterion then requires both switches to be triggered at the same time. Hence, a signal of fall will be given only when the above discussed falling criteria are met for both directions at the same time. Practically, this can be achieved by designing the two MEMS switches connected in series as will be discussed next.

## 4. Design of a Switch Triggered by Human Fall

### 4.1. The Sensor Concept

In this section, we focus on designing a MEMS switch that is triggered by the acceleration thresholds extracted from the previous section. A Single-Degree-Of-Freedom (SDOF) model is utilized. The idea here is to design a switch that works based on the pull in phenomenon due to the effect of the electrostatic and acceleration from fall [33–36]. When the acceleration signal affects the proof mass of a cantilever beam, it leads to pull-in instability, where the proof mass hits the electrode. This action closes an electric circuit, which can be utilized for alarming purposes. To cover most of the fall scenarios in multiple possible directions, multiple chips are electrically connected to each other. Each chip contains two switches connected in series, one with an upper stationary electrode and the other with a lower stationary electrode. These chips and switches are placed on the same location on the body within one simple package that forms the whole detection system.

**Figure 9.**
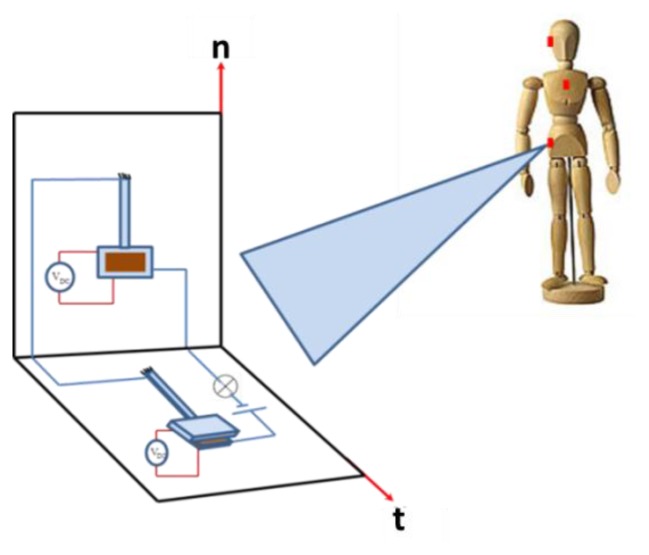
A schematic of the proposed sensor in FF or BF.

Figure 9 shows a schematic of the proposed sensor. It consists of two acceleration threshold switches connected in series. These are electrostatically biased capacitive switches, which are composed of a flexible electrode that deflects with acceleration, separated by a small distance from a stationary electrode. The principle of operation for the sensor depends on the normal and tangential components of the fall acceleration. When the sensor is subjected to a fall signal of acceleration components exceeding the thresholds specified in Table 4, both switches will close the circuit shown in Figure 9, which can be used for alarming purposes. Exceeding the threshold by one component, on the other hand, will not close the circuit and no fall signal will be triggered.

**Figure 10.**
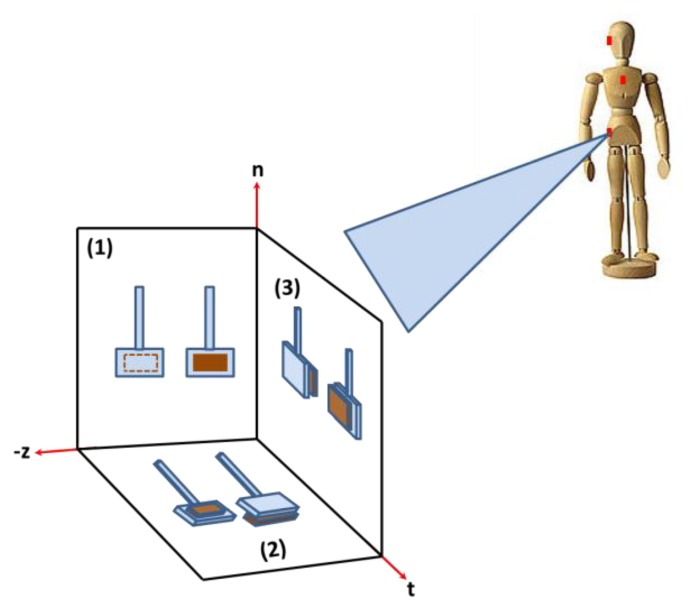
A schematic of the proposed sensor for any direction.

The arrangement of switches shown in Figure 9 triggers a forward fall event. For more generic cases and to trigger any kind of fall in any direction, additional switches required as shown in Figure 9. For example, in the case of a side fall, switches on chips number 2 and 3 will be affected by the fall acceleration, where the negative z-axis in the global coordinate will be the tangential axis in the local coordinate system. Hence, the number of switches in Figure 9 is increased from 2 switches to 6 to cover any kind of fall of any direction as shown in Figure 10. In this arrangement, each pair of switches is assigned for one of the three planes of fall (forward, backward, and side). In each pair, one switch will have its stationary electrode either placed on the substrate (switch 1) or placed on top of the proof mass (switch 2).

**Figure 11.**
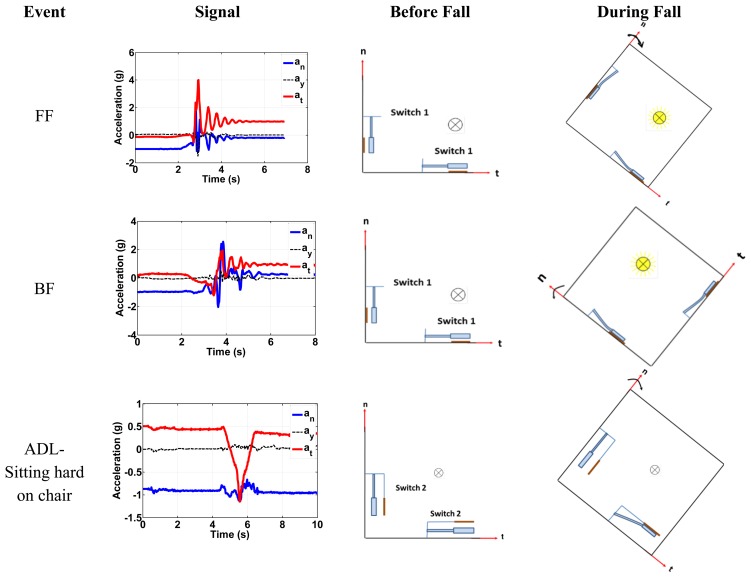
The switch response under falls and ADL signals for the chest position.

Figure 11 shows the switching action for the forward fall (FF), a backward fall (BF), and one of the ADLs for the chest position. First row shows the switches pull in due to the forward fall event with acceleration magnitude exceeding the minimum threshold. Switch 1 will be triggered by normal component of the fall signal, while switch 2 will be triggered by the tangential components of the fall signal. For the alarm to go on (confirming fall event), both inertia switches should be pulled in when the fall acceleration exceeds the thresholds limits. Both switches are connected in series. The second row is the BF with both switch 1 types used to trigger the fall event. The third row shows how triggering only one component will indicate an ADL or a non-fall event since only the normal component exceeds the fall limit. If we consider the FF case in Figure 11, first row, the normal component has a positive value of 2.9 g. This positive normal component will base-excite switch 1 causing a downward deflection of its proof mass toward the substrate causing pull-in. The tangential component also has a positive value of 4 g. This signal will base-excite switch 1 causing a deflection of its proof mass toward the stationary electrode also causing it to pull-in. Table 7 presents a summary for the kinds of switches needed for all fall scenarios.

### 4.2. A Single-Degree-of-Freedom Model of the Switch

**Figure 12.**
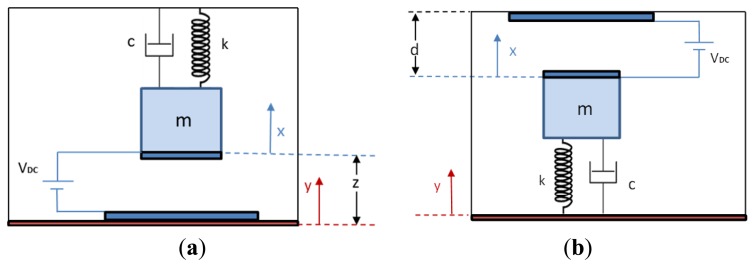
A model of a parallel plate capacitor switch for lower and upper electrode positions, (**a**) lower (switch 1); (**b**) upper (switch 2).

The design details of the individual switches of the previous section are discussed here. Each switch is modeled as a SDOF parallel plate capacitor subjected to a base excitation. For each switch, the position of the electrode with respect to the base motion needs to be taken into account to distinguish between the directions of the normal and tangential accelerations for different types of falls. Figure 12a shows a schematic of the model of switch 1 of the parallel plate capacitor of lower stationary electrode when subjected to the base motion. Figure 12b shows a schematic of switch 2 of a parallel plate capacitor with upper stationary electrode when subjected to the base motion.

The relative acceleration of the mass with respect to the base is expressed as *z̈* = *ẍ* − *ÿ*, where *ẍ*(*t*) is the acceleration of the mass and *ÿ*(*t*) is the acceleration of the base. In this model the base acceleration is the acceleration that comes from the falling or ADL. Hence, we express the term *ÿ* in terms of gravity g and acceleration amplitude *a*(*t*). The general governing equations are written for normal and tangential direction, respectively, as follows:

(3) $$m_{\text{eff}}\ddot{z}+cż+kz+ma_{n}(t)g\pm \frac{ɛAV_{DC}^{2}}{2{\left(d\pm z\right)}^{2}}+mg\cos[\theta (t)]=0$$

(4) $$m_{\text{eff}}\ddot{z}+cż+kz+ma_{t}(t)g\pm \frac{ɛAV_{DC}^{2}}{2{\left(d\pm z\right)}^{2}}+mg\sin[\theta (t)]=0$$

where, *m* is the mass of the beam, *k* is the spring stiffness, *c* is the damping constant, and the term with direct current voltage *V_DC_* is the electrostatic force, *ε* is the dielectric constant of air, which equals 8.85 × 10^−12^ (*C*^2^/*Nm*^2^), *A* is the area of the electrode, *d* is the gap width between the stationery electrode and flexible electrode connected to the mass. The positive sign of the electrostatic force is for switch 1, and the negative sign is for switch 2. The angle *θ(t)* is given by:

(5) $$\theta (t)=\tan^{-1}\left(\frac{a_{t}(t)}{a_{n}(t)}\right)$$

The fall signal is approximated as a point load acting at the tip of the cantilever beam. The effective mass and stiffness for a cantilever beam with mass at the tip according to *Younis* [39] are given by:

(6) $$m_{\text{eff}}=0.236m;\;k=\frac{3EI}{L^{3}}$$

where *E* is the modulus of elasticity, *I* is the second moment of inertia, and *L* is the total length of the cantilever beam.

### 4.3. Case Study

**Figure 13.**
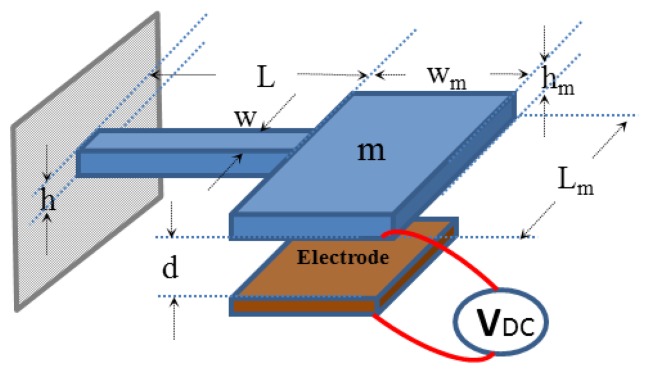
Schematic of the proposed switch.

Different measured fall signals will be imported to the analytical model to study the performance of the switch. The design parameters for the cantilever beam and the proof mass are shown in Figure 13.

Table 8 shows the design parameters for the switch. Note here that the beam thickness is different from the proof mass thickness, which indicates that such a switch needs to be fabricated using bulk micromachining techniques. According to these dimensions, the stiffness of the cantilever beam has a value of 6.4, while the pull in voltage without the effect of the acceleration is calculated, using the pull-in formula of a spring-mass system [39], as 2.2 V. The natural frequency is estimated around 1130.7 Hz.

**Figure 14.**
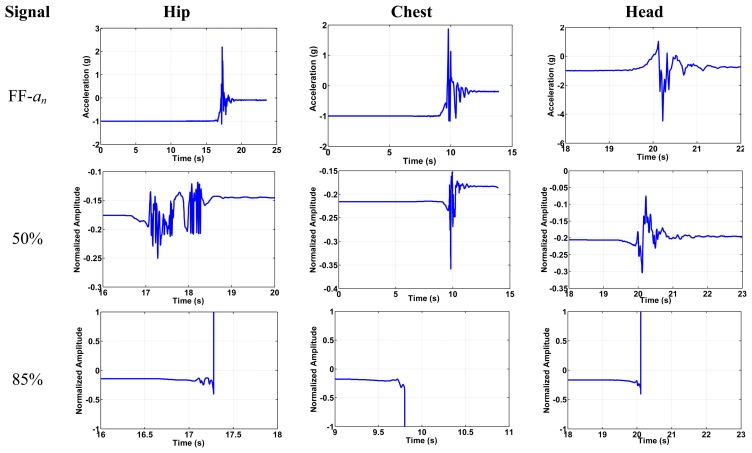
Simulation of the MEMS switch under the effect of 50% of fall signal (representing the ADL) and 85% of fall signal (representing the start of the fall threshold).

Next, we show simulation results for the response of the device under the effect of the dynamic acceleration signals imported from the collected experimental data for the three kinds of fall in the normal and tangential directions. The results are obtained using long-time integration of the equation of motion, Equations (3) and (4), using the Runge–Kutta method. Some of the results are shown in Figure 14.

In the figure, the amplitude response is normalized with respect to the gab width of the switch. Hence, an absolute normalized deflection equal to one means that the proof mass has hit the stationary electrode indicating pull-in. Figure 14 shows the imported signal of the normal acceleration with the effect of 50% of the fall signal, which as discussed is considered representation of the ADL signal. As shown, the switch stays in the stable position in this case. The third row shows the response of the switch to the 85% of the fall signal, where the switch pulls in due to loss of stability. This means that the proof mass hits the electrode at a voltage of 1.6 V. Without the signal, the pull in voltage is around 2.18 V for the hip position, while it pulls in at 1.6 V and 1.7 V for chest and head positions, respectively. After simulating all fall signals for the three different positions, we summarize the results for the pull in voltage of each fall signal in Table 9.

The pull-in voltage at zero acceleration (*V_Pull_*[*a*(0)]), and the pull-in voltage with fall acceleration (*V_Pull_*[*a*(0)]) are the minimum voltages causing pull-in using long-time integration approach and assuming zeros initial conditions. The last column in Table 9 represents a measurement of the accuracy of the sensor to distinguish between the falls and ADL. A percentage close to 100% means that any small acceleration will highly give false alarm of fall event, while a percentage close to 0% means that even a true fall signal may not affect the sensor and no alarm given. Of course these results are for a single switch only. As discussed in Section 4.2, through the use of multiple switches connected electrically in series, the possibility of false positives and wrong responses are minimized.

Results in Table 9 cover the pull-in voltage values for a specific human body, with specific height and mass distribution mentioned in Table 2. Those parameters are different from one person to another, and to achieve a good performance for the sensor, the pull-in voltage should be adjusted according to those parameters.

One final note is regarding the effect of damping on the proposed switch. Particularly, because of the close distance between the proof mass and the substrate and the big surface area of the proof mass, squeeze-film damping (SQFD) can have dominant effect on the device [40,41]. However, since the natural period of the proposed device is 1/1130.7 Hz = 8.85 × 10^−4^*s* is much lower than the period between the successive peaks of the input acceleration signal (typically of order 0.1–0.5 s). Hence the device experiences the acceleration pulse as a quasi-static signal, which makes it insensitive to SQFD and the variation of damping in general.

## 5. Summary and Conclusions

We have presented detailed experimental investigation on the kinematics of the human body during falls as observed by uni-axial accelerometers. A tri-axial accelerometer has been used to analyze the Activities of Daily Living and the forward, backward and side falls for the hip, chest and head positions. We analyzed the data and derived guidelines and thresholds that can be used for fall detection. To minimize false positives, due to the potential overlap between ADLs and fall signals, we proposed using multi inertia-activated switches connected in series. We showed that 2 inertia switches at least are needed to detect a specific kind of fall, one for each acceleration component. It was shown that no ADL, excluding the jumping and running activity signals, has any effect on the stability of the switch.

We believe that further studies are needed to address issues such as the sensitivity and reliability of the detection method for various kinds of falls and for a variety of human body specifications (different weights, heights, *etc.*). Also, the proposed devices need to be fabricated and tested in real-life fall scenarios to verify the concept and to reveal other practical constraints.

## Figures and Tables

**Table 1.** Normal and tangential accelerations equations for hip, chest and head positions.

***a***	**Hip**	**Chest**	**Head**

*a_n_*	−*a_y_* sin *β* − *a_x_* cos *β* – *g* sin *β*	−*a_y_* sin *γ* − *a_x_* cos *γ* – *g* sin *γ*	−*a_y_* sin *γ* − *a_x_* cos *γ* – *g* sin *γ*
*a_t_*	−*a_y_* cos *β* + *a_x_* sin *β* – *g* cos *β*	+*a_y_* cos *γ* + *a_x_* sin *γ* + *g* cos *γ*	+*a_y_* cos *γ* + *a_x_* sin *γ* + *g* cos *γ*

**Table 2.** Parameter values for two-link model.

**Parameter**	**Value**
*m*	85 kg
*m_1_*	42% of *m*
*m_2_*	58% of *m*
*L*	1.8 m
*L_1_*	60% of *L*
*L_2_*	40% of *L*

**Table 3.** Acceleration limits.

**Position**	**Limit (g)**	

	**Normal**	**Tangential**
Hip	3.2	5.5
Chest	1.9	2.9
Head	3.5	3.6

**Table 4.** Threshold accelerations (85% of the fall peaks) for hip, chest and head.

**Falls**	***a***	**Hip**		**Chest**		**Head**	
		
		**Peak**	**Threshold**	**Peak**	**Threshold**	**Peak**	**Threshold**
FF	*a_n_*	3.2	2.7	2.9	2.5	3.5	3
	*a_t_*	5.5	4.7	4	3.4	4.8	4.1

BF	*a_n_*	4.6	3.9	3.6	3.1	4.9	4.2
	*a_t_*	6.8	5.8	1.9	1.6	3.6	3.1

SF	*a_n_*	4	3.4	5.5	4.7	4.8	4.1
	*a_t_*	6.6	5.6	5.2	4.4	4	3.4

**Table 5.** ADL as a 50% of the fall peaks.

**Position**	**Hip**	**Chest**	**Head**
Maximum ADL peak	1.5	1.7	1.75
Minimum fall peak	3.2	2.9	3.5
% (Max/Min)	47%	57%	50%

**Table 6.** 50% of the fall peaks.

**Falls**	***a***	**Hip**	**Chest**	**Head**
FF	*a_n_*	1.6	1.45	1.75
	*a_t_*	2.75	2	2.4

BF	*a_n_*	2.3	1.8	2.45
	*a_t_*	3.4	0.95	1.8

SF	*a_n_*	2	2.75	2.4
	*a_t_*	3.3	2.6	2

**Table 7.** Switch types needed for each fall scenario.

**Position**	**Fall**	***a***	**Switch Type**
Hip	FF	*a_n_*	Switch 1
		*a_t_*	Switch 2

	BF	*a_n_*	Switch 1
		*a_t_*	Switch 1

	SF	*a_n_*	Switch 1
		*a_t_*	Switch 1

Chest	FF	*a_n_*	Switch 1
		*a_t_*	Switch 1

	BF	*a_n_*	Switch 1
		*a_t_*	Switch 1

	SF	*a_n_*	Switch 2
		*a_t_*	Switch 1

Head	FF	*a_n_*	Switch 2
		*a_t_*	Switch 2

	BF	*a_n_*	Switch 1
		*a_t_*	Switch 1

	SF	*a_n_*	Switch 1
		*a_t_*	Switch 1

**Table 8.** Design parameters for the proposed switch.

**Parameters**	**Cantilever Beam**				**Proof Mass**		
	
	**L (μm)**	**h (μm)**	**w (μm)**	**d (μm)**	**L_m_(μm)**	**h_m_(μm)**	**w_m_(μm)**
Value	1000	10	160	2	600	150	600

**Table 9.** Pull in voltage for each fall signal. * pull in voltage due to the electrostatic force without the effect of the fall acceleration, (*V_Pull_*[*a*(0)]) = 2.18 *V*. ** pull in voltage due to the electrostatic force with the fall acceleration.

**Fall Signal**		***V****_Pull_***[*a*(0)]** **	$\left(\overset{V_{\text{Pull}}\left[a(t)\right]}{\;}/\underset{*V_{\text{Pull}}\left[a(0)\right]}{\;}\right)\%$

Hip	*FF_an_*	1.6	73
	*FF_at_*	0.7	32
	*BF_an_*	1.4	64
	*BF_at_*	0.5	23
	*SF_an_*	1.3	60
	*SF_at_*	0.5	23

Chest	*FF_an_*	1.6	73
	*FF_at_*	1.2	55
	*BF_an_*	1.3	60
	*BF_at_*	1.5	69
	*SF_an_*	1.5	69
	*SF_at_*	0.6	28

Head	*FF_an_*	1.7	78
	*FF_at_*	1.7	78
	*BF_an_*	1.1	50
	*BF_at_*	1.1	50
	*SF_an_*	1.1	50
	*SF_at_*	1.3	60

## Appendix A

The kinetic of energy of the system of Figure 1 *T* and its potential energy *V* can be written as:

(A1) $$T=\frac{1}{2}m_{2}\left[L_{1}^{2}{\dot{\theta }}^{2}+\frac{L_{2}^{2}}{4}{\dot{\phi }}^{2}+L_{1}L_{2}\dot{\theta }\dot{\phi }\cos(\theta -\phi )\right]+\frac{1}{6}m_{1}L_{1}^{2}{\dot{\theta }}^{2}$$

(A2) $$V=\left(\frac{1}{2}m_{1}+m_{2}\right)L_{1}g\cos\theta +\frac{1}{2}m_{2}gL_{2}\cos\phi$$

Accordingly, the Lagrangian can be expressed as:

(A3) $$\ell =\frac{1}{2}m_{2}\left[L_{1}^{2}{\dot{\theta }}^{2}+\frac{L_{2}^{2}}{4}{\dot{\varphi }}^{2}+L_{1}L_{2}\dot{\theta }\dot{\varphi }\cos(\theta -\varphi )\right]+\frac{1}{6}m_{1}L_{1}^{2}{\dot{\theta }}^{2}-\left(\frac{1}{2}m_{1}+m_{2}\right)L_{1}g\cos\theta -\frac{1}{2}m_{2}gL_{2}\cos\phi$$

Applying the Lagrange equation for the two generalized coordinates *θ* and *ϕ* yields the following coupled equations of motion:

(A4) $$\left(\frac{1}{3}m_{1}+m_{2}\right)L_{1}^{2}\ddot{\theta }+\frac{1}{2}m_{2}L_{1}L_{2}[\ddot{\phi }\cos(\theta -\phi )+{\dot{\phi }}^{2}\sin(\theta -\phi )]-\left(\frac{1}{2}m_{1}+m_{2}\right)L_{1}g\sin\theta =0$$

(A5) $$\frac{1}{2}m_{2}L_{1}L_{2}[\ddot{\theta }\cos(\theta -\phi )-{\dot{\theta }}^{2}\sin(\theta -\phi )]+\frac{1}{24}m_{2}L_{2}^{2}\ddot{\phi }-\frac{1}{2}m_{2}L_{2}g\sin\phi =0$$

Equations (A4) and (A5) are integrated numerically in time, using Runge–Kutta scheme and their results are used for the formulas in Table 1.
